# A 4 Mb High Resolution BAC Contig on Bovine Chromosome 1q12 and Comparative Analysis With Human Chromosome 21q22

**DOI:** 10.1002/cfg.476

**Published:** 2005-06

**Authors:** Cord Drögemüller, Anne Wöhlke, Tosso Leeb, Ottmar Distl

**Affiliations:** 1 Institute for Animal Breeding and Genetics University of Veterinary Medicine Hannover Germany; 2 Institute for Animal Breeding and Genetics University of Veterinary Medicine Hannover Bünteweg 17p Hannover 30559 Germany

## Abstract

The bovine RPCI-42 BAC library was screened to construct a sequence-ready ~4 Mb single contig of 92 BAC clones on BTA 1q12. The contig covers the region between the genes *KRTAP8P1* and *CLIC6*. This genomic segment in cattle is of special interest as it contains the dominant gene responsible for the hornless or polled phenotype in cattle. The construction of the BAC contig was initiated by screening the bovine BAC library with heterologous cDNA probes derived from 12 human genes of the syntenic region on HSA 21q22. Contig building was facilitated by BAC end sequencing and chromosome walking. During the construction of the contig, 165 BAC end sequences and 109 single-copy STS markers were generated. For comparative mapping of 25 HSA 21q22 genes, genomic PCR primers were designed from bovine EST sequences and the gene-associated STSs mapped on the contig. Furthermore, bovine BAC end sequence comparisons against the human genome sequence revealed significant matches to HSA 21q22 and allowed the *in silico* mapping of two new genes in cattle. In total, 31 orthologues of human genes located on HSA 21q22 were directly mapped within the bovine BAC contig, of which 16 genes have been cloned and mapped for the first time in cattle. In contrast to the existing comparative bovine–human RH maps of this region, these results provide a better alignment and reveal a completely conserved gene order in this 4 Mb segment between cattle, human and mouse. The mapping of known polled linked BTA 1q12 microsatellite markers allowed the integration of the physical contig map with existing linkage maps of this region and also determined the exact order of these markers for the first time. Our physical map and transcript map may be useful for positional cloning of the putative polled gene in cattle. The nucleotide sequence data reported in this paper have been submitted to EMBL and have been assigned Accession Numbers AJ698510–AJ698674.


**Comparative and Functional Genomics**
*Comp Funct Genom* 2005; **6**: 194–203.
Published online in Wiley InterScience (www.interscience.wiley.com). **DOI:** 10.1002/cfg.476

**Short Communication**

### A 4 Mb high resolution BAC contig on bovine chromosome 1q12 and comparative analysis with human chromosome 21q22

Cord Drögemüller*[#], Anne Wöhlke[#], Tosso Leeb and Ottmar Distl
*Institute for Animal Breeding and Genetics, University of Veterinary Medicine, Hannover, Germany*

**Correspondence to: Cord Drögemüller, Institute for Animal Breeding and Genetics, University of Veterinary Medicine Hannover, Bünteweg 17p, 30559 Hannover, Germany. E-mail: Cord.Droegemueller@tiho-hannover.de*

*[#] These authors contributed equally to this work..*

### Abstract

**The bovine RPCI-42 BAC library was screened to construct a sequence-ready ∼4 Mb single contig of 92 BAC clones on BTA 1q12. The contig covers the region between the genes *KRTAP8P1* and *CLIC6*. This genomic segment in cattle is of special interest as it contains the dominant gene responsible for the hornless or polled phenotype in cattle. The construction of the BAC contig was initiated by screening the bovine BAC library with heterologous cDNA probes derived from 12 human genes of the syntenic region on HSA 21q22. Contig building was facilitated by BAC end sequencing and chromosome walking. During the construction of the contig, 165 BAC end sequences and 109 single-copy STS markers were generated. For comparative mapping of 25 HSA 21q22 genes, genomic PCR primers were designed from bovine EST sequences and the gene-associated STSs mapped on the contig. Furthermore, bovine BAC end sequence comparisons against the human genome sequence revealed significant matches to HSA 21q22 and allowed the *in silico* mapping of two new genes in cattle. In total, 31 orthologues of human genes located on HSA 21q22 were directly mapped within the bovine BAC contig, of which 16 genes have been cloned and mapped for the first time in cattle. In contrast to the existing comparative bovine–human RH maps of this region, these results provide a better alignment and reveal a completely conserved gene order in this 4 Mb segment between cattle, human and mouse. The mapping of known polled linked BTA 1q12 microsatellite markers allowed the integration of the physical contig map with existing linkage maps of this region and also determined the exact order of these markers for the first time. Our physical map and transcript map may be useful for positional cloning of the putative polled gene in cattle. The nucleotide sequence data reported in this paper have been submitted to EMBL and have been assigned Accession Numbers AJ698510–AJ698674.** **Copyright © 2005 John Wiley & Sons, Ltd.**

Received: 5 July 2004
Revised: 3 February 2005
Accepted: 17 March 2005

**Keywords:** **BTA 1; HSA 21; BAC; contig; comparative mapping; polled; cattle**

### Introduction

A bovine physical map consisting of a contiguous assembly of overlapping BAC clones (contig) is considered a necessary prerequisite for the accurate assembly of whole genome shotgun sequences in the current efforts to obtain the bovine genome sequence (Gibbs *et al.*, 2002). Although construction of preliminary genome-wide BAC contigs for cattle (*Bos taurus*) is in progress (Larkin *et al.*, 2003; Schibler *et al.*, 2004), there is a need to construct highly accurate physical maps of targeted regions to facilitate targeted sequencing and the discovery of species specific genes or quantitative trait loci (QTL) affecting economically important traits. Currently, successful positional cloning studies using detailed contig maps of specific cattle genome regions have been rare, e.g. the identification of the bovine *LIMBIN* gene causing dwarfism in Japanese brown cattle (Takeda *et al.*, 2002;

Copyright © 2005 John Wiley & Sons, Ltd.

Takeda and Sugimoto, 2003) or the analysis of the bovine *DGAT1* gene as a functional candidate for milk yield and composition (Grisat *et al.*, 2002, 2004; Winter *et al.*, 2002, 2004).

In cattle, the hornless or polled phenotype is of special interest due to its economical importance in beef production. Hornless individuals are much safer to work with and they are less likely to injure themselves or other animals. The bovine polled phenotype shows a monogenic autosomal dominant inheritance and the still-unknown gene has been genetically mapped to the centromeric region of bovine chromosome (BTA) 1 (Georges *et al.*, 1993; Schmutz *et al.*, 1995; Harlizius *et al.*, 1997). The first cattle–human comparative maps have been determined at low resolution by chromosome painting experiments and revealed that the proximal part of BTA 1 shows conserved synteny with human chromosome (HSA) 21 (Threadgill *et al.*, 1991; Chowdhary *et al.*, 1996). The recent expansion in the available number of bovine ESTs (Smith *et al.*, 2001), in combination with sequence information of the nearly finished human genome project, provided the resources for detailed comparative maps. Subsequently, a medium-resolution bovine-human whole genome comparative map was generated by RH-mapping (Band *et al.*, 2000). Additionally, different comparative RH maps of the centromeric BTA 1 region were constructed but revealed inconsistencies concerning the existence of chromosomal rearrangements between BTA 1q12 and HSA 21q22 (Rexroad *et al.*, 1999, 2000; Drögemüller *et al.*, 2002). Considering the difficulties with high-resolution RH mapping, a successful comparative positional cloning strategy of the polled gene should be complemented by a precise clone-based physical map of this region.

Herein we describe the construction of a BAC contig covering a ~4 Mb segment on BTA 1q12 and its comparative analysis with the syntenic region on HSA 21q22, which has previously been shown to contain the polled mutation. This genomic contig integrates a large number of genes and markers of physical, genetic, cytogenetic and RH maps of BTA 1q12. As a first step towards positional cloning of the polled gene in cattle, this high-resolution BAC contig map represents a valuable resource for future fine mapping and sequencing efforts.

### Materials and methods

### DNA library screening and chromosome walking

Library screenings with cDNA clones were performed as described (Drögemüller *et al.*, 2002). PCR-amplified DNA fragments were labelled with $^{32}P$ and hybridized as probes on the high-density clone filters of the bovine genomic BAC library RPCI-42 (Warren *et al.*, 2000) according to the RPCI protocol (**http://www.chori.org/bacpac/**). BAC DNA was prepared from 100 ml overnight cultures using the Qiagen Midi plasmid kit according to the modified protocol for BACs (Qiagen, Hilden, Germany). Insert sizes were determined as described (Martins-Wess and Leeb, 2003).

### DNA sequence analysis

Isolated BAC DNA was sequenced with the thermosequenase kit (Amersham Biosciences, Freiburg, Germany) and a LICOR 4200L automated sequencer. BAC DNA was sequenced with IRD-labelled T7 and Sp6 sequencing primers. Sequence data were analysed with Sequencher 4.1.4 (GeneCodes, Ann Arbor, MI, USA). BLAST database searches were performed at NCBI (**http://www.ncbi.nlm.nih.gov/**) for human mRNA alignments against bovine EST entries and for the bovine–human comparison against the whole human genome sequence (build 34.3). Repetitive elements were identified with the RepeatMasker searching tool (**http://www.repeatmasker.org/**). Single-copy sequences were used to design primer pairs for the chromosome walking, using the program GeneFisher (**http://bibiserv.techfak.uni-bielefeld.de/genefisher/**).

### Results

To construct a BAC contig of the bovine polled gene region we started to screen a bovine BAC library by hybridization of 12 different heterologous human IMAGE cDNA clones (Table 1). The physical localizations of six representative gene associated BAC clones were established by RH mapping and FISH on BTA 1q12 (Drögemüller *et al.*, 2002) prior to the beginning of a chromosome walking strategy. Further sequence tagged

Copyright © 2005 John Wiley & Sons, Ltd. 

**Table 1.** Human cDNA hybridization probes within the bovine BAC contig

| Human gene symbol | IMAGE-ID | RZPD clone ID |
|---|---|---|
| *TIAM1* | 3 197 030 | IMAGp 998 O157814 |
| *SOD1* | 436 140 | IMAGp 998 B131026 |
| *HUNK* | 768 063 | IMAGp 998 H161890 |
| *C21orf108* | 25 729 | IMAGp 998 G19138 |
| *C21orf59* | 124 398 | IMAGp 998 E07121 |
| *SYNJ1* | 2 038 462 | IMAGp 998 M235017 |
| *OLIG2* | 2 170 611 | IMAGp 998 P045361 |
| *IL10RB* | 842 859 | IMAGp 998 E042085 |
| *GART* | 2 901 218 | IMAGp 998 J037162 |
| *SON* | 1 696 332 | IMAGp 998 N134307 |
| *KCNE2* | 2 308 895 | IMAGp 998 A245722 |
| *DSCR1* | 324 006 | IMAGp 998 B07734 |

site (STS) probes that allowed the gradual joining of the individual emerging contigs into one large contig were generated from the BAC end sequences obtained from appropriate clones. Overlaps between clones were determined by STS content analysis. In total, 109 new STS markers were generated (Table 2). The complete BAC contig consisted of 92 clones (Figure 1). The physical mapping information derived from the contig assembly was refined by taking into account estimated BAC insert sizes from pulsed-field gels. The average insert size of the 92 BAC clones was 162 kb (range 30–200 kb). The entire contig spans approximately 4 Mb and can be covered with a minimal tiling path of 32 clones (Figure 1).

The clone-based physical map was anchored to the linkage and RH map of BTA 1 by STS content mapping of five previously described bovine microsatellites (*ARO9, ARO24, TGLA49, SOD1MICRO2, BM6438*) and two EST markers (EST0601, EST1413) (Figure 1). During construction of the bovine contig, primers were designed for 25 HSA 21q22 genes from corresponding bovine EST sequences (Table 3). PCR analysis of all 92 BAC clones with the gene-specific EST primer pairs revealed positive clones and the localization of these genes on the contig (Figure 1).

In total, 165 BAC end sequences with an average read length of 726 bp, totalling approximately 120 kb of genomic survey sequences, were generated. Thus, the BAC end sequences cover approximately 3% of the genomic region under study. The sequence information of these 165 BAC ends has been deposited in the EMBL nucleotide database under Accession Numbers AJ698510–AJ698674. Sequence alignments revealed eight pairs of identical BAC ends. The end sequences contain an average GC content of 44.3%, marginally exceeding the value of 41% that is generally accepted as the average GC content in mammalian genomes (Lander *et al.*, 2001). The GC content analysis further suggests that BTA 1q12 is indeed closely related to HSA 21q22, which has a GC content of 43.2% in the corresponding 4 Mb region. An analysis of repetitive sequences revealed that 39.1% of the BAC end sequences consisted of bovine repetitive DNA, mainly LINE (18.9%) and SINE (14.9%) elements; only 3.4% were of retroviral origin (LTRs) and 1.3% represented DNA transposons. In 56 cases, all or the majority of the BAC end sequences represented repetitive sequences and were therefore discarded for STS design. The repeat masked BAC end sequences were subjected to BLAST comparisons against the sequence of the human genome (build 34.3). The matches obtained confirmed the homology between the cloned chromosomal region in cattle with HSA 21q22. Significant and unique matches (e-value $<10^{-5}$) against human genomic sequences were observed for 38 (23%) bovine BAC end sequences. All but one of the 38 matches mapped to the expected location on HSA 21q22 (Table 4). All these BLAST matches corresponded well with the overall clone order in the bovine BAC contig and confirmed the correct assembly. In some cases the BLAST searches revealed the presence of genes within BAC end sequences and confirmed the previously obtained mapping results (Table 4). The *C21orf62* and *SFRS15* genes could be localized *in silico* by this approach on the contig for the first time (Figure 1). Only one single sequence (380C19-SP6) matched to a different human chromosome during the BLAST search. This unexpected BLAST result probably indicates a chimeric clone, as this BAC has been anchored in the contig by 4 STS markers and a gene specific bovine EST primer pair (Figure 1).

In total, the construction of this contig confirms the mapping of 15 previously mapped BTA 1 genes and provides 16 new chromosomal assignments of bovine orthologues to the human genes *SFRS15, C21orf45, C21orf108, C21orf63, C21orf59, C21orf66, C21orf62, IFNGR2, C21orf4, SON, MRPS6, C21orf82, C21orf51, KCNE1, DSCR1* and *CLIC6*. The gene order of the 31 assigned genes in the bovine BAC clone

Copyright © 2005 John Wiley & Sons, Ltd. 

**Table 2.** Primer sequences of all used STS markers belonging to BAC end sequences of RPCI-42 clones

| STS marker | Forward primer sequence (5′–3′) | Reverse primer sequence (5′–3′) | $T_M$ (°C) | PCR product (bp) |
|---|---|---|---|---|
| 383K23-SP6 | ATCTGAGCCACCAGAAAAGTC | GCATATGTCTTGGGAACATG | 56 | 257 |
| 383K23-T7 | CTTCTTTCCCAAGGACATAGTG | TTCCTGAGCACTCTCTTTATGG | 58 | 184 |
| 394A5-SP6 | ACTCAGAGGGCAATTGTAGAAG | GTTGCCAGCAGGGAATGG | 58 | 593 |
| 386F4-T7 | CCTGTCCCACCAGAAAGC | TGATGAACAGGGTAAGTTGG | 55 | 252 |
| 352O20-SP6 | TCCTGTTATATACCCCTCATGC | GAAGGGGGGAACAGTTATTGAG | 59 | 337 |
| 44B5-T7 | GGAGAATGGATACACAAGGTTC | CCTTCTCAAAAGGGGAAATACG | 58 | 395 |
| 394A5-T7 | CTCTCATGTTGGTAAGTGAGAG | CTTTGCTGTCCACTTGCAAC | 57 | 415 |
| 292J15-T7 | TCAGTGTGAATTTGCCCCATG | GTCATCTTTGGTGATCTCTC | 54 | 308 |
| 234N12-T7 | TCAATGGCCAAAGGATTCAC | GTAAACGGTAATGCCTTTCC | 56 | 318 |
| 352O20-T7 | ACTGACACTTTTCTGGGAGTAG | TCAATGGCCAAAGGATTCAC | 57 | 390 |
| 506K17-SP6 | AGGGTGTAAGTCTTCAGAAGAC | CTAAATCTTATCCAGGGCCTTG | 58 | 265 |
| 506K17-T7 | GATCCCCATGATGGTGCATC | CACAGAAGTTTTAGGTGGACAG | 58 | 193 |
| 506K15-SP6 | TTACCTAGGGGTGGTTTTTCAG | TATCCACATCACAGCCAAGATC | 58 | 327 |
| 292J15-SP6 | TCCCCTCAGCCTCCAGAAG | CACCAGGGAAGTTCTGGATC | 58 | 259 |
| 311D23-T7 | CAACCTACAATCGCATCATCC | GAGAACAGGTGAAGGGAGAG | 57 | 420 |
| 320O18-T7 | AATGTATCCTCCCTAAGGACAG | GGAGATAGAGAAAGCTTCTGAC | 57 | 265 |
| 506K15-T7 | CAGGAGGCTGTTAAACTTTGTG | ATAGTCCCCTTCTTCGATTACG | 58 | 520 |
| 320O18-SP6 | AATTCTTCAGGTGGAGAGTGTC | AAACCTGCCCCCTATCTAAGC | 59 | 342 |
| 311D23-SP6 | CCATCATTGAGGTCAGGGTTG | GCGGCAGCTATAACCACAAG | 59 | 517 |
| 447G24-T7 | GCTGGTTATTCTATCCCCTCTC | TTGTCTTGCCCAATGGTGAC | 58 | 193 |
| 301M9-T7 | CTGCCTTTTGCATAGGTGAAG | GGGGAAGGGGCTAATTTAGAC | 58 | 414 |
| 26I16-T7 | CCCTTTTTCCTCTCTGCCTTC | ACTAGCTGGGAAAAGACATTGC | 59 | 517 |
| 180G7-T7 | CTCAGGCTTTCCTTCTCCAA | GGACAGGAAGCTGACGTTT | 59 | 161 |
| 196M18-T7 | CCTTGTCTTCATCATCTGATCG | CCACCTTGCCTCCTCTCTC | 58 | 275 |
| 374D19-T7 | AGGAAGGGGTAAGACTCTTGTG | TTCTAGTTAGCCTGTACGTTGC | 59 | 375 |
| 301M9-SP6 | GACATGACTGAAGTGACTTAGC | GAGGAGGGAGGATACTAGAGAG | 60 | 521 |
| 447G24-SP6 | AAGCATCCCAAACTGTAAGC | AAAAGCCTAACTTGGGAAGG | 56 | 415 |
| 199N3-T7 | CCTAAATTCCTTTGGCTCTTCC | CCCTACCCTAGAGTGACCATG | 59 | 267 |
| 266O23-SP6 | AACAGCCAGGGGTTCTGAC | GGTTTCCTGAGAGGTCACATC | 59 | 320 |
| 316N2-T7 | GTCGGTAACACACGCACATC | ACCCTATGACATCATCTGTTGC | 59 | 310 |
| 196M18-SP6 | TCTGGTTGTACGTTGGTGATG | CTGTGCCCCTAACTAATAAAGC | 58 | 245 |
| 292J17-SP6 | CTCCAGAGAAGTTCTGTGTCTG | GAGGAATCTCGGGAGATTGC | 60 | 200 |
| 420A17-SP6 | CTTCTCGCCCACTCTATCTC | AGAGATTCAGTTCAGACGTCTG | 57 | 326 |
| 46I17-SP6 | AGAGTGCTGGCCAGATGTC | TCACCATGTTCTGCTTTGAC | 57 | 520 |
| 553A8-SP6 | TCGGTATCACACTTCTGTACAC | TCTCTCACTTTTCCTTCCTCTC | 59 | 300 |
| 266O23-T7 | ACATATACTGGCAGGCCTCTG | TGGAAGCCCTACTGGTCAAG | 59 | 393 |
| 199N3-SP6 | CAAGGTTAGTTTGGGAACAAGG | GTGATGCTGGCCACCAAC | 58 | 518 |
| 213N17-T7 | ACACTTCTACACTCTCACAAGG | CTTGTTAGTCTGACCCGTGAG | 58 | 478 |
| 46I17-T7 | TTGCAGGCAGTTTCCTTACTG | CCTTTAGGATGGACTGGTTGG | 59 | 470 |
| 493P3-T7 | ACTTGGAACCTAAGAGAGGATG | CCTGGGATGACTGAGAAGAG | 57 | 520 |
| 553A8-T7 | AATCAAGGTCTCCATGTGTAGC | TACGAGGTACAAACTCAGGTTC | 59 | 217 |
| 320F13-T7 | TTCAAATCTGTCCTCCACATCC | TGAGAATGCATCAGAGAGAGTG | 59 | 185 |
| 68K7-T7 | CATCAGTATCCTTTCAGCAACC | GGCAGAGAACAGGCATTCAG | 58 | 225 |
| 249E18-T7 | CCCCACAGCACTATTTCTTGG | TATAGCAGGAGGCATCAAAAGC | 59 | 190 |
| 320F13-SP6 | CCCATGGAATTTCCCTGCTAG | TGCATACCAGTCTGCAAGTTC | 59 | 475 |
| 493P3-SP6 | AGGAGTTAGTGACAGACACTTG | GAAGAGACTGGTGGACATCC | 58 | 520 |
| 271E18-SP6 | CGATGACTCACTTTGCTGTAC | GGAAACAGGAAATGAGGTTGC | 58 | 184 |
| 470N12-SP6 | CACAAAAGCAAGCAGTTCTCTC | TGTGTAGTGTACCATTGGCAAG | 59 | 452 |
| 249E18-SP6 | GGGTGAGTCCAGGGAGATG | AATGAGGGGCACAGCAGAG | 60 | 206 |
| 217G23-SP6 | TGGCCTTCCGTGTTTTCAC | TCACTGGTTTAGACTCAGTG | 54 | 317 |
| 271E18-T7 | GAAGCTGTCATCCTCTTTTTGG | AAAAGCGGCTAAGGGAACAC | 59 | 500 |
| 569F23-SP6 | TGACTTTGTTAGCTGGCTCAG | CTTCTGTTTCATTGTGGGTCTG | 59 | 322 |
| 518G6-SP6 | CCATCCCTGTCATGTTTTGG | TACAGGCACAGACTCATCAG | 57 | 458 |
| 538E7-SP6 | CTGTGGCCTACATAGTTTAGAG | GGTTTCATAGAGTCCCCTGATG | 58 | 179 |
| 470N12-T7 | AGGATCCTGCAAGCTGTGC | CTGACAGCAGGAACTTTTC | 58 | 522 |
| 569F23-T7 | CTGCCGGATCTGTGATTTGC | TTCTGCCTTTCCTCCTTTAACC | 59 | 163 |

Copyright © 2005 John Wiley & Sons, Ltd. 

**Table 2.** Continued

| STS marker | Forward primer sequence (5′–3′) | Reverse primer sequence (5′–3′) | $T_M$ (°C) | PCR product (bp) |
|---|---|---|---|---|
| 217G23-T7 | GGAGGTTTTAGGAAAAGGATGC | ACTGCAGGTGAACTCTTTACC | 57 | 230 |
| 161B10-SP6 | CCGTCACTCCTTGTTTATCTTG | GTCAAGATTTTGTCAGCCCATC | 59 | 188 |
| 518G6-T7 | GAATTTGGGGGGAAGTGTAGC | CTCACTGCGGGATATTGATTCC | 59 | 521 |
| 76J4-T7 | CCCTGCAAGCAACAAAAGTG | TCCTCAATCCCACCCTCTTG | 59 | 279 |
| 219G21-T7 | ACAAGAAACAGAGTGCTTGG | TTTCACCAAACTCACCTAGC | 56 | 310 |
| 76J4-SP6 | TCAGGCTTAGGTGATCTCATCC | GGCAAAGTTCTCAATCCAAAGG | 59 | 369 |
| 21K5-T7 | AACGGTACAAGGAGAAAAGG | TGTCATAAATCCTGGGTCAC | 55 | 464 |
| 554P19-SP6 | CTTTACTCCTCGTAGCTGTC | TTCTGTGAGGGCAGAGTG | 56 | 368 |
| 219G21-SP6 | CTTAGAAGTGTGGCCGGTAG | GTGTTGATAAACTGACCCTCTG | 58 | 418 |
| 351B8-SP6 | CATGAATACTTAACACTACTG | CCTCTAATGTGGAATCCAG | 52 | 147 |
| 52K19-T7 | AAATGCAGGACAGAGAGAATCG | GTTATGTCCTAGGGCTGTGTC | 59 | 279 |
| 554P19-T7 | GGAATGGGTAGCTGTTCC | CCATAGAGTCCCAAAGAGTC | 54 | 160 |
| 552B21-T7 | TTCTTCAAAACCCACTCCTTCC | AAACTACACCCGGTCCTCTTC | 59 | 244 |
| 52K19-SP6 | ATCCCCAGCCAAGTGTAGTC | TGCCACTGACAGAATGAATCTG | 59 | 180 |
| 487A22-T7 | ACGCTTACTGAGGAAGGATGG | CAGGAAGGGGGACAGATACG | 59 | 363 |
| 564N14-T7 | CCTACAATGCTCTCAGCTGTC | GAGAAAGCTTGCTCATGTTGAC | 60 | 474 |
| 241F8-SP6 | ACAGACCTAAGTCTAGCTTG | TTCCCTGATGAAAGAGATGC | 55 | 368 |
| 552B21-SP6 | TCTATTACCTGGTTTCGGTTGG | GAAACTGAGCTCTTACTGCAG | 59 | 143 |
| 332I5-SP6 | GAGCAATGTAATATTGACTGG | ATTCCCCTCCCCAAATTTACC | 58 | 184 |
| 79M3-T7 | TATCTCCTTGAGGTTTGGAACG | GGGTCGTACACTGAATAAGTAG | 58 | 311 |
| 564N14-SP6 | AGTGACTAACACGCACGTTG | GCTAGTTCCTTGCCCTATTGTC | 60 | 450 |
| 534N15-T7 | ATCGGTGAACTTTCCTCATTCC | GCCAATATCACAGCCATTTCC | 59 | 358 |
| 368A9-T7 | GTTCCTATCAGCTCCGATTCG | GGCTATGGGTCTGGTAAATGG | 59 | 410 |
| 543J10-T7 | TCATTAGCACTGCCAGTTCTTC | CCAGGAGAGGGCAAATTCAAC | 59 | 348 |
| 79M3-SP6 | TAAACTAGCTGAGCAAGCCAAG | GCACATTAAAGTGGCTGGAAC | 59 | 520 |
| 534N15-SP6 | ATCACTGTTAGGTGACAGGTTG | ATGGTCACTGGTCCACACAG | 59 | 419 |
| 79N19-SP6 | TCTCTTCTCATCCCTGGGAAC | GGCACCTGGTATCTCTTATGC | 59 | 273 |
| 204M10-SP6 | GTTTACACCGTGGCTTTAGC | TTCATGCTGTTTGCTGAACC | 58 | 452 |
| 218J7-SP6 | AGAAATGGCCGTGATCTGTG | CATCAGCCTACAGAACATAACG | 58 | 308 |
| 221H19-T7 | GGTTGAGAGAAGAAGGGCTTG | CAGAGAAAGCAAAGCTGAGAAG | 59 | 143 |
| 79N19-T7 | AGGGATGACATAACCATAGG | CTTGCTGTTATGTCACAACG | 54 | 115 |
| 204M10-T7 | ATAACAGACCAGCGGGTGAC | TTCTACAAAGACCACAGCCATC | 59 | 209 |
| 218J7-T7 | TGCAGGTGGCTCTTCAGTATC | AGGTAGGGAGCCTGGATTGT | 60 | 154 |
| 380B9-SP6 | TTCGTAGTCTCTAAGGGAAG | CTCTTGGCCTTTATCAAGTC | 53 | 313 |
| 420O24-T7 | CACCTTCTTCCAAGGCTAGTG | AGGTTACTCTGTCTCTGAATGC | 59 | 269 |
| 167I16-T7 | CTCAGGGAATGATTCTTTCC | CCATCCAACCATCTCATC | 55 | 253 |
| 51I7-SP6 | AGAAGTCTAATAAGCTCTGCTGCAT | AACAAACGTTTCCCCTCTACA | 59 | 100 |
| 182B8-T7 | TTTTTCTAGTTCTGTGTATTC | ACCTCTCTTAAATGTAGAC | 50 | 408 |
| 382D7-SP6 | TGGCCTGTGACTAGTTTAGTTC | CATGGGTATGAAAACCACAGTG | 58 | 159 |
| 420O24-SP6 | GGTTGCTATAGCAGCCTCTC | GAATGCCCTAGACGTCCATC | 58 | 367 |
| 167I16-SP6 | CATCCCTGAAGGCTTTAGG | TCTTATTGAGCACCCACTG | 55 | 397 |
| 182B8-SP6 | CTTCTCCAGCGGATCTTC | GACGGAAGGTTTTGTTACC | 54 | 517 |
| 80B9-T7 | AAGCATCCCATCCCAGGAAC | TATCTGTCTCTCTGGGCATCC | 59 | 521 |
| 132D12-SP6 | TTAAGGATGAGGGGGTCTAGG | CTTCAGGGAAATGGGCTCTC | 58 | 269 |
| 540F4-T7 | GTTGGTAGAAAAAGCCACCATC | CCCCATAAGCAGCACTTCTC | 59 | 310 |
| 31K20-T7 | AGCTTCAGTTGAACCCAAGTAC | CTTCCAGTAGTTCACCAGACTG | 59 | 422 |
| 543J23-SP6 | ATTCTGAATTCAGGCCAACC | TGTTTACAGCAACAGCTGTC | 56 | 199 |
| 80B9-SP6 | AAGGGCACAGGAGATTTTCAAG | AAAGAGGCTGGGCTGAGATG | 59 | 312 |
| 328M7-T7 | AGGAATGGCAGGGAACTGAC | CACACATATAGCATGTGCTTGG | 59 | 183 |
| 31K20-SP6 | GTGTCAGTTCTGTGGGTTTCC | GAGGTCCAGGTCCTTCCTTC | 59 | 268 |
| 494B13-SP6 | GGTACATTGGAGTCTCTGACAC | TGATGGAGGCAAAACAGGTTC | 59 | 281 |
| 543J23-T7 | GGAGCAGTCTGCTATCAAAGG | GTCATATCAACAGAGTGCATGC | 59 | 232 |
| 494B13-T7 | TAGATAACAGAGGGTGGGGATG | TAACTCAGCTCTGATGTGGTAC | 58 | 181 |

Copyright © 2005 John Wiley & Sons, Ltd. 

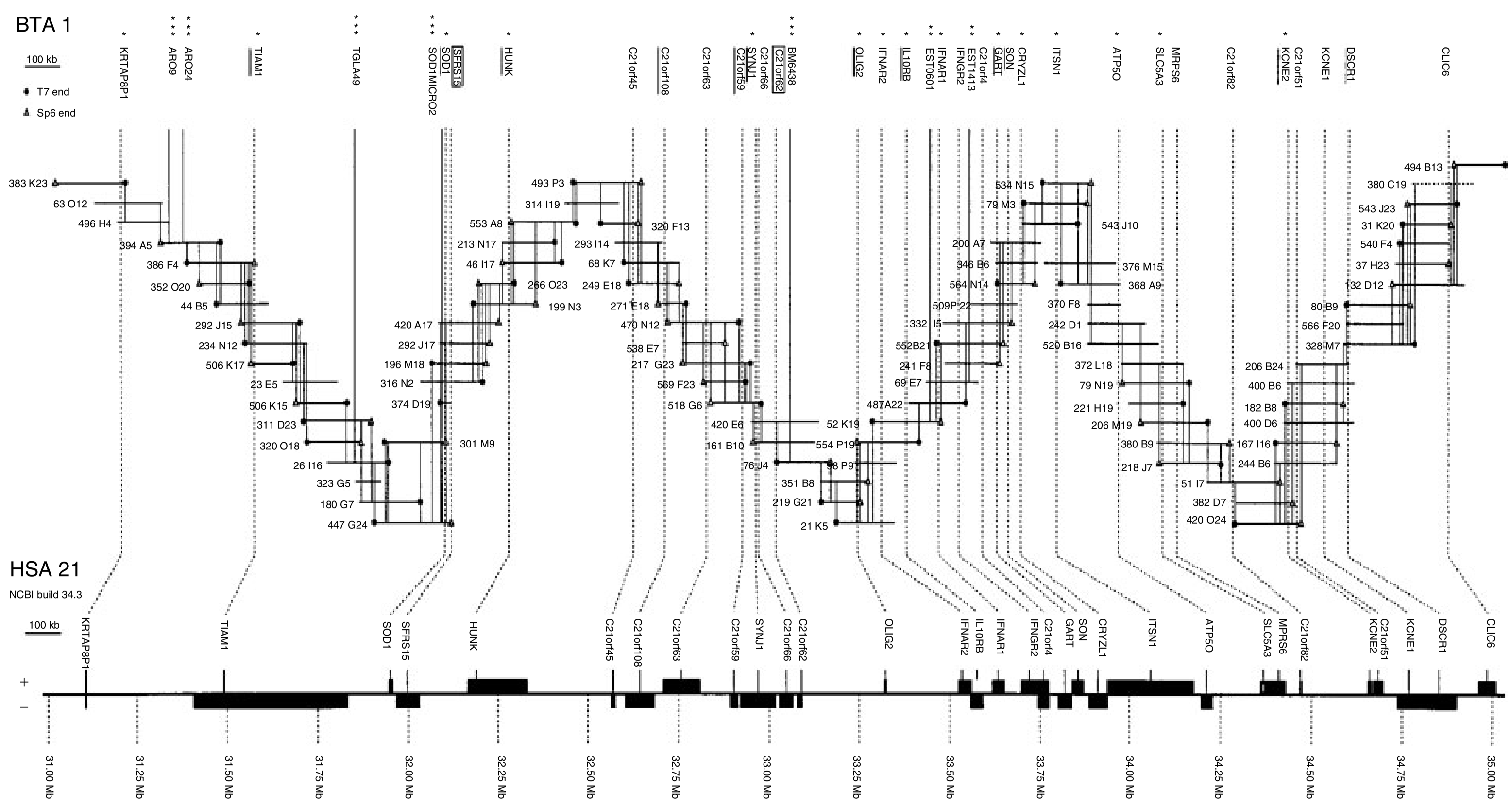

**Figure 1.** Physical map of the isolated bovine BAC contig on BTA 1q12. All mapped loci are indicated vertically at the top. Previously published BTA 1 mapping results are marked by one (genes), two (ESTs) or three (microsatellites) asterisks. Underlined gene markers were initially assigned by human cDNA hybridization probes. The two framed genes were localized on the contig *in silico*. RPCI-42 BAC clones are shown below the markers as continuous horizontal lines with their corresponding abbreviated clone names. A single chimeric BAC is shown by a dashed horizontal line. A minimal tiling path of 32 clones is indicated by thick lines. Bovine microsatellite, EST and STS markers are represented by vertical solid lines. Bovine markers that are associated to corresponding human genes are plotted by dotted vertical lines and linked to 31 genes on the 4 Mb sequence segment of HSA 21q22 (NCBI build 34.3) at the bottom. Comparative mapping of 31 gene-associated markers revealed a complete conservation of the gene order across the entire 4 Mb interval between *Bos taurus* and *Homo sapiens*

**Table 3.** Gene-specific bovine EST primer sequences within the BAC contig

| Human gene symbol | Bovine EST (Accession Nos) | Forward primer sequence (5′–3′) | Reverse primer sequence (5′–3′) | $T_M$ (°C) | PCR product (bp) |
|---|---|---|---|---|---|
| *KRTAP8P1* | X98351 | TTGCTGAAATACCAGAGGCA | ATGACAAGAGTCATGAGCATGG | 55 | 212 |
| *TIAM1* | BE757612 | GCACTGGAAAGCAAATTACC | AAAATCACCACACCTCACTC | 55 | 509 |
| *SOD1* | M81129 | GTTTGGCCTGTGGTGTAATTGGAA | GGCCAAAATACAGAGATGAATGAA | 60 | 275 |
| *C21orf45* | BE668325 | GGAAGATGTTTTGAAAGCC | GAATGTGGGCCTTGGAAC | 60 | 101 |
| *C21orf63* | BM107239 | CTTGGTCATCAGAGTGTCATG | GTGTCCAAACCACTGTTCATC | 60 | 277 |
| *C21orf59* | BI537216 | CGTCATCAAAGAATCAGAGG | CCCACGTAGTCTGAAAGCTTC | 60 | 81 |
| *SYNJ1* | BE752169 | GGTCCTAGTCACTGGATG | GTGGGCATTAAGACTCAG | 60 | 205 |
| *C21orf66* | AW462169 | GTGGGACCCCTTTTCTAC | CTGCATTCACCACTGAAGG | 60 | 89 |
| *IFNAR2* | AV666571 | CCCTTCACCAACCCCTCTAC | TCCTCCCCAGGGAGAACAC | 59 | 227 |
| *IFNAR1* | X68443 | AGAAGTTTTCGTCGTCCTTTG | TGATGGTGGTATTCAGGTTCTTC | 55 | 290 |
| *IFNGR2* | BF654282 | CCCGTTCAGGAGTAACTTCA | GTTTCACAGCAAGATATGTTGC | 57 | 117 |
| *C21orf4* | BF043420 | GTGCTCGAGTTTGGCTCTTC | CATAGGCACCAAAGAGAATCC | 59 | 84 |
| *GART* | BF039462 | CAGTGAAGAGGGGTGACACTG | GCCATTTTCTCCAAGCCGTAC | 60 | 122 |
| *SON* | BF041673 | GATAAATGGAAGCGCTTACCAG | CTGATTACAGGCAAGTGTTAC | 58 | 515 |
| *CRYZL1* | BG692873 | GGACAGAAGCTGCTGGAACC | CTTGCTCCGTCCATTATCAG | 59 | 109 |
| *ITSN1* | AV605000 | TCAAAAGAGCCTTAGAAG | GAAAATATCAATCTCCTG | 60 | 108 |
| *ATP50* | BM364861 | GCCACCTGTTCAGATATATGGC | CTACTCGCAACAACTCCTTCTC | 60 | 110 |
| *SLC5A3* | BE664959 | GTGGCCCTGTATTTTATCCTG | CCACCAAATCGCTTGGACAAG | 60 | 321 |
| *MRPS6* | BI775325 | CTTGGTGGACTTTTATGCAC | GGACGATCCCTTCACATTC | 60 | 137 |
| *C21orf82* | CB444814 | CAAGGCTGCAAATTCAGAGG | TAGTGTCTTGGCCTGGGTTC | 60 | 183 |
| *KCNE2* | BG938225 | CAGGACGGAAATATGCCAAC | GATTTCACCGTGCTCACCAG | 60 | 234 |
| *C21orf51* | BM433498 | CTGCTGCTGTATATCTTGGC | GCTTCCTCTTCTCAGCTTCC | 60 | 124 |
| *KCNE1* | BE486735 | CTTCTTCACCCTGGGCATCATG | CAGCCAGCTGGTTCTCAATGAC | 60 | 179 |
| *DSCR1* | BF041330 | CCCTCTTTAGGACTTATGAC | CAGTCTTATGTAGCTGGAG | 60 | 128 |
| *CLIC6* | CB456208 | CCGAGCATATGCATTGTTCAAG | TCACGAGGACCATCTGTGATG | 59 | 320 |

contig (Figure 1) corresponds exactly to the gene order of the NCBI HSA 21q22 map (**http://www.ncbi.nlm.nih.gov/mapview/**; build 34.3), which lists 50 gene loci in the interval between *KRTAP8P1* and *CLIC6*. Of these 50 loci, seven represent computer predicted hypothetical genes and five are pseudogenes, while 38 genes have at least some experimental evidence. The physical size of the investigated region and the distances between the mapped genes seems to be conserved between human and cattle. A high degree of gene order conservation can also be observed with respect to annotated murine genes. Some of the mapped bovine genes are assigned to the linkage map of mouse chromosome (MMU) 16. The current NCBI sequence map of MMU 16 (**http://www.ncbi.nlm.nih.gov/mapview/**; build 32.1) lists 19 of the 31 analysed genes in a similar order as in cattle or human.

### Discussion

Here we describe a ∼4 Mb single BAC contig that is predicted to contain the putative bovine polled gene. It establishes the physical order of the genetic microsatellite markers from different linkage maps that define the linked region and enables an exact determination of the candidate interval size. The physical map described here has a higher resolution and accuracy than other currently available maps, which often have conflicting data with respect to marker order (Rexroad *et al.*, 1999, 2000; Drögemüller *et al.*, 2002). The recombination frequency could not be reliably estimated in the investigated region, as there were inconsistencies between the different genetic maps of the BTA 1 centromere (Taylor *et al.*, 1998). The markers *TGLA49* and *BM6438* that are separated by 0.3 cM on the current MARC cattle linkage map (**http://www.marc.usda.gov**) are separated by roughly 1.4 Mb and the recombination frequency would be approximately 0.2 cM/Mb. This low value for the recombination frequency seems reasonable, considering that the investigated region is located close to the centromere, where low recombination frequencies have to be expected. The precise physical assignment of the linked microsatellites will benefit future efforts towards

Copyright © 2005 John Wiley & Sons, Ltd. 

**Table 4.** Significant (e-value $<10^{-5}$) and unique BLAST matches of bovine RPCI-42 BAC clone end sequences against human genomic sequences (build 34.3)

| Query | HSA | Human gene symbol | Alignment start | Strand | E-value | Bitscore |
|---|---|---|---|---|---|---|
| 496H4-T7 | 21 | | 31 061 111 | + | 7e-16 | 91.7 |
| 496H4-SP6 | 21 | | 31 184 614 | − | 1e-41 | 176 |
| 386F4-SP6 | 21 | *TIAM1* | 31 412 535 | − | 9e-24 | 117 |
| 506K17-T7 | 21 | *TIAM1* | 31 514 415 | − | 1e-07 | 63.9 |
| 311D23-SP6 | 21 | *TIAM1* | 31 773 134 | − | 2e-40 | 172 |
| 301M9-T7 | 21 | *TIAM1* | 31 813 620 | + | 4e-32 | 145 |
| 374D19-T7 | 21 | | 31 950 932 | + | 7e-09 | 67.9 |
| 374D19-SP6 | 21 | *SFRS15* | 31 978 365 | − | 0 | 769 |
| 447G24-SP6 | 21 | *SFRS15* | 31 978 365 | − | 0 | 777 |
| 266O23-SP6 | 21 | | 32 067 314 | + | 1e-14 | 87.7 |
| 46I17-SP6 | 21 | | 32 139 591 | + | 8e-06 | 58 |
| 213N17-SP6 | 21 | | 32 139 591 | + | 7e-06 | 58 |
| 213N17-T7 | 21 | *HUNK* | 32 294 724 | − | 1e-23 | 117 |
| 46I17-T7 | 21 | | 32 319 376 | − | 6e-16 | 91.7 |
| 44B5-T7 | 21 | | 32 533 995 | + | 5e-14 | 85,7 |
| 249E18-SP6 | 21 | | 32 702 372 | − | 3e-08 | 65.9 |
| 68K7-SP6 | 21 | | 32 702 372 | − | 4e-08 | 65.9 |
| 569F23-T7 | 21 | *SYNJ1* | 32 978 627 | − | 1e-79 | 303 |
| 161B10-SP6 | 21 | | 33 022 790 | + | 1e-07 | 63.9 |
| 518G6-T7 | 21 | *C21orf66* | 33 053 865 | − | 6e-31 | 141 |
| 76J4-T7 | 21 | *C21orf62* | 33 095 281 | + | 3e-17 | 95.6 |
| 21K5-T7 | 21 | | 33 399 040 | + | 3e-14 | 85.7 |
| 564N14-T7 | 21 | *GART* | 33 796 824 | + | 1e-60 | 240 |
| 241F8-SP6 | 21 | *GART* | 33 804 431 | − | 2e-06 | 60 |
| 534N15-T7 | 21 | *ITSN1* | 34 053 681 | + | 8e-15 | 87.7 |
| 543J10-T7 | 21 | *ITSN1* | 34 117 826 | − | 2e-06 | 60 |
| 79M3-SP6 | 21 | *ITSN1* | 34 118 696 | − | 2e-09 | 69.9 |
| 372L18-T7 | 21 | | 34 297 245 | + | 1e-57 | 230 |
| 204M10-SP6 | 21 | | 34 316 613 | + | 2e-09 | 69.9 |
| 221H19-SP6 | 21 | | 34 316 613 | + | 2e-09 | 69.9 |
| 204M10-T7 | 21 | | 34 481 575 | − | 6e-07 | 61.9 |
| 400B6-T7 | 21 | | 34 629 490 | + | 1e-54 | 220 |
| 400D6-T7 | 21 | | 34 629 496 | + | 4e-51 | 208 |
| 400B6-SP6 | 21 | *DSCR1* | 34 846 334 | − | 5e-94 | 351 |
| 400D6-SP6 | 21 | *DSCR1* | 34 846 334 | − | 5e-94 | 351 |
| 543J23-SP6 | 21 | *DSCR1* | 34 899 686 | + | 2e-16 | 93.7 |
| 37H23-SP6 | 21 | *CLIC6* | 35 009 095 | − | 2e-29 | 135 |
| 380C19-SP6 | 13 | | 57 544 793 | + | 4e-09 | 67.9 |

the positional cloning of the bovine polled gene, as the precise marker position with respect to coding genes is now available. The BAC contig we have generated also represents a resource for the isolation of additional polymorphic markers for fine mapping efforts.

In this study three techniques were used to localize bovine genes on the contig. During the first phase of contig construction we applied a comparative approach. The recent availability of the complete sequence and gene catalogue of the long arm of HSA 21 (Hattori *et al.*, 2000) has facilitated the procedure, using appropriate human heterologous screening probes to isolate bovine BAC clones. In the second phase of contig construction we increased the marker density by exploiting the available bovine EST resources that allowed the generation of bovine gene-specific primers for bovine orthologues of human genes. To develop these primers we used the rapidly growing bovine EST sequence information in combination with data on exon/intron boundaries from the human genome. Finally, in some cases genes could be localized on the contig *in silico*

Copyright © 2005 John Wiley & Sons, Ltd. 

according to the BLAST search results of BAC end sequences. Using these three approaches, 31 genes could be assigned to the BAC contig, of which the following 15 gene loci had previously been mapped to cattle chromosome 1 with low precision: *KRTAP8P1* (Harlizius *et al*., 1997); *SOD1, IFNAR1, IFNAR2* (Threadgill *et al*., 1991); *GART* (Chowdhary *et al*., 1996); *ATP50* (Smith *et al*., 2001); *SLC5A3* (Rexroad *et al*., 1999); *TIAM1, HUNK, SYNJ1, OLIG2, IL10RB, KCNE2* (Drögemüller *et al*., 2002); *ITSN1* (Laurent *et al*., 2000);. *CRYZL1* (Stone *et al*., 2002), respectively.

This bovine–human comparative map provides the highest resolution comparative map of HSA 21q22 with the centromeric region of BTA 1 reported to date. The analysis of gene content of the investigated genomic region on BTA 1q12 revealed perfect synteny conservation between cattle and human. In contrast to the current bovine RH maps (Rexroad *et al*., 1999, 2000; Drögemüller *et al*., 2002), we found no evidence for the existence of chromosomal rearrangements in cattle, which is in part due to recent changes in the human genome assembly. High overall gene order conservation can also be observed with respect to the mouse. In other studies different gene orders within conserved synteny groups were observed across mammalian species (Schibler *et al*., 1998). One possible explanation for the strong conservation observed here could be that the high gene content of BTA 1q12 interfered with major chromosome rearrangements during mammalian evolution.

In conclusion, the BAC contig we have constructed is an essential preliminary step toward the targeted positional cloning of the bovine polled gene. The mapping information that we present here will facilitate the accurate assembly of whole-genome shotgun DNA sequences of this region during the upcoming cattle genome project.

### Acknowledgements

We thank Pieter de Jong and his lab for providing the RPCI-42 library. We would also like to thank Heike Klippert-Hasberg for excellent DNA sequencing. This study was supported by a grant from the German Research Council (DFG) (DI 333/8-1).

### References

Band MR, Larson JH, Rebeiz M, *et al.* 2000. An ordered comparative map of the cattle and human genomes. *Genome Res* **10**: 1359–1368.

Chowdhary BP, Fronicke L, Gustavsson I, Scherthan H. 1996. Comparative analysis of the cattle and human genomes: detection of ZOO-FISH and gene mapping-based chromosomal homologies. *Mamm Genome* **7**: 297–302.

Drögemüller C, Bader A, Wöhlke A, *et al.* 2002. A high-resolution comparative RH map of the proximal part of bovine chromosome 1. *Anim Genet* **33**: 271–279.

Georges M, Drinkwater R, King T, *et al.* 1993. Microsatellite mapping of a gene affecting horn development in *Bos taurus*. *Nat Genet* **4**: 206–210.

Gibbs R, Weinstock G, Kappes S, *et al.* 2002. Bovine genomic sequencing initiative; **http://www.genome.gov/10002154**

Grisart B, Coppieters W, Farnir F, *et al.* 2002. Positional candidate cloning of a QTL in dairy cattle: identification of a missense mutation in the bovine *DGAT1* gene with major effect on milk yield and composition. *Genome Res* **12**: 222–231.

Grisart B, Farnir F, Karim L, *et al.* 2004. Genetic and functional confirmation of the causality of the *DGAT1* K232A quantitative trait nucleotide in affecting milk yield and composition. *Proc Natl Acad Sci USA* **101**: 2398–2403.

Harlizius B, Tammen I, Eichler K, Eggen A, Hetzel DJS. 1997. New markers on bovine chromosome 1 are closely linked to the polled gene in Simmental and Pinzgauer cattle. *Mamm Genome* **8**: 255–257.

Hattori M, Fujiyama A, Taylor TD, *et al.* 2000. The DNA sequence of human chromosome 21. *Nature* **405**: 311–319.

Lander ES, Linton LM, Birren B, *et al.* 2001. Initial sequencing and analysis of the human genome. *Nature* **409**: 860–921.

Larkin DM, Everts-van der Wind A, Rebeiz M, *et al.* 2003. A cattle-human comparative map built with cattle BAC-ends and human genome sequence. *Genome Res* **13**: 1966–1972.

Laurent P, Elduque C, Hayes H, *et al.* 2000. Assignment of 60 human ESTs in cattle. *Mamm Genome* **11**: 748–754.

Martins-Wess F, Leeb T. 2003. Increased throughput of BAC/PAC insert size determinations by stacking gels during pulsed-field gel electrophoresis. *Biotechniques* **34**: 718–720.

Rexroad CE, Schlapfer JS, Yang Y, Harlizius B, Womack JE. 1999. A radiation hybrid map of bovine chromosome one. *Anim Genet* **30**: 325–332.

Rexroad CE, Owens EK, Johnson JS, Womack JE. 2000. A 12000 rad whole genome radiation hybrid panel for high resolution mapping in cattle: characterization of the centromeric end of chromosome 1. *Anim Genet* **31**: 262–265.

Schibler L, Vaiman D, Oustry A, Giraud-Delville C, Cribiu EP. 1998. Comparative gene mapping: a fine-scale survey of chromosome rearrangements between ruminants and humans. *Genome Res* **8**: 901–915.

Schibler L, Roig A, Mahe MF, *et al.* 2004. A first generation bovine BAC-based physical map. *Genet Sel Evol* **36**: 105–122.

Smith TP, Grosse WM, Freking BA, *et al.* 2001. Sequence evaluation of four pooled-tissue normalized bovine cDNA libraries and construction of a gene index for cattle. *Genome Res* **11**: 626–630.

Copyright © 2005 John Wiley & Sons, Ltd. 

Schmutz SM, Marquess FL, Berryere TG, Moker JS. 1995. DNA marker-assisted selection of the polled condition in Charolais cattle. *Mamm Genome* **6**: 710–713.

Stone RT, Grosse WM, Casas E, *et al*. 2002. Use of bovine EST data and human genomic sequences to map 100 gene-specific bovine markers. *Mamm Genome* **13**: 211–215.

Takeda H, Takami M, Oguni T, *et al*. 2002. Positional cloning of the gene *LIMBIN* responsible for bovine chondrodysplastic dwarfism. *Proc Natl Acad Sci USA* **99**: 10 549–10 554.

Takeda H, Sugimoto Y. 2003. Construction of YAC/BAC contig map for the BTA 6q21 region containing a locus for bovine chondrodysplastic dwarfism. *Anim Biotechnol* **14**: 51–59.

Taylor JF, Eggen A, Aleyasin A, *et al*. 1998. Report of the first workshop on the genetic map of bovine chromosome 1. *Anim Genet* **29**: 228–235.

Threadgill DS, Kraus JP, Krawetz SA, Womack JE. 1991. Evidence for the evolutionary origin of human chromosome 21 from comparative gene mapping in the cow and mouse. *Proc Natl Acad Sci USA* **88**: 154–158.

Warren W, Smith TP, Rexroad CE, *et al*. 2000. Construction and characterization of a new bovine bacterial artificial chromosome library with 10 genome-equivalent coverage. *Mamm Genome* **11**: 662–663.

Winter A, Kramer W, Werner FA, *et al*. 2002. Association of a lysine-232/alanine polymorphism in a bovine gene encoding acyl-CoA : diacylglycerol acyltransferase (DGAT1) with variation at a quantitative trait locus for milk fat content. *Proc Natl Acad Sci USA* **99**: 9300–9305.

Winter A, Alzinger A, Fries R. 2004. Assessment of the gene content of the chromosomal regions flanking bovine *DGAT1*. *Genomics* **83**: 172–180.

Copyright © 2005 John Wiley & Sons, Ltd. 
